# Establishment of Hepatitis C Virus RNA-Replicating Cell Lines Possessing Ribavirin-Resistant Phenotype

**DOI:** 10.1371/journal.pone.0118313

**Published:** 2015-02-20

**Authors:** Shinya Satoh, Kyoko Mori, Youki Ueda, Hiroe Sejima, Hiromichi Dansako, Masanori Ikeda, Nobuyuki Kato

**Affiliations:** Department of Tumor Virology, Okayama University Graduate School of Medicine, Dentistry, and Pharmaceutical Sciences, 2-5-1 Shikata-cho, Okayama 700-8558, Japan; University of Missouri-Columbia, UNITED STATES

## Abstract

**Background:**

Ribavirin (RBV) is a potential partner of interferon-based therapy and recently approved therapy using direct acting antivirals for patients with chronic hepatitis C. However, the precise mechanisms underlying RBV action against hepatitis C virus (HCV) replication are not yet understood. To clarify this point, we attempted to develop RBV-resistant cells from RBV-sensitive HCV RNA-replicating cells.

**Methodology/Principal Findings:**

By repetitive RBV (100 μM) treatment (10 weeks) of 3.5-year-cultured OL8 cells, in which genome-length HCV RNA (O strain of genotype 1b) efficiently replicates, dozens of colonies that survived RBV treatment were obtained. These colonies were mixed together and further treated with high doses of RBV (up to 200 μM). By such RBV treatment, we successfully established 12 RBV-survived genome-length HCV RNA-replicating cell lines. Among them, three representative cell lines were characterized. HCV RNA replication in these cells resisted RBV significantly more than that in the parental OL8 cells. Genetic analysis of HCV found several common and conserved amino acid substitutions in HCV proteins among the three RBV-resistant cell species. Furthermore, using cDNA microarray and quantitative RT-PCR analyses, we identified 5 host genes whose expression levels were commonly altered by more than four-fold among these RBV-resistant cells compared with the parental cells. Moreover, to determine whether viral or host factor contributes to RBV resistance, we developed newly HCV RNA-replicating cells by introducing total RNAs isolated from RBV-sensitive parental cells or RBV-resistant cells into the HCV RNA-cured-parental or -RBV-resistant cells using an electroporation method, and evaluated the degrees of RBV resistance of these developed cells. Consequently, we found that RBV-resistant phenotype was conferred mainly by host factor and partially by viral factor.

**Conclusions/Significance:**

These newly established HCV RNA-replicating cell lines should become useful tools for further understanding the anti-HCV mechanisms of RBV.

## Introduction

Hepatitis C virus (HCV) infection causes persistent hepatitis, leading often to liver cirrhosis and hepatocellular carcinoma [1, 2]. Since approximately 170 million people are estimated to be infected with HCV worldwide, this infection is a serious global health problem [3]. HCV is an enveloped virus with a positive single-stranded 9.6 kilobase (kb) RNA genome and belongs to the *Flaviviridae* family. HCV encodes a single open reading frame, producing a large polyprotein precursor of approximately 3000 amino acids (aa). This precursor polyprotein is processed by host and virus proteases into the following mature proteins: core, envelope 1 (E1), E2, p7, nonstructural protein 2 (NS2), NS3, NS4A, NS4B, NS5A, and NS5B [4, 5].

Ribavirin (RBV) is a synthetic guanosine analog and shows efficacy in the treatment of viral diseases. RBV has been used in combination with pegylated-interferon (PEG-IFN) in the former standard therapy for patients with chronic hepatitis C. This treatment achieves greater than 50% sustained virological response (SVR), while monotherapy with IFN achieves only a 30% SVR [6]. Furthermore, by combining PEG-IFN and RBV with a direct-acting antiviral (DAA), such as telaprevir or boceprevir, more than 70% of treatment-naïve patients recently showed an SVR [7–9]. Very recently, many DAAs for HCV infection have been developed, and newer treatments using these DAAs, such as sofosbuvir, simeprevir, or sofosbuvir plus ledipasvir, were approved by the FDA [10].

To date, several mechanisms underlying RBV antiviral action against HCV have been proposed [11, 12]: the inhibition of NS5B RNA-dependent RNA polymerase activity, the induction of mutagenesis in the HCV genome leading to a so-called “error catastrophe”, the enhancement of the IFN-signaling pathway, the inhibition of inosine monophosphate dehydrogenase (IMPDH) leading to GTP depletion, and immunomodulation of the switching of the Th cell phenotype from type 2 to type 1. Although most of these mechanisms were proposed based on studies using HuH-7 (human hepatoma cell line)-derived cells, which are currently used as the only cell culture system for robust HCV replication, the effective concentrations (50–1000 μM) of RBV were much higher than the clinically achievable concentrations [11, 13, 14]. Indeed, the effective concentration of RBV in our HuH-7-derived cell assay system (OR6) [15], in which genome-length HCV RNA (O strain of genotype 1b) encoding renilla luciferase replicates efficiently, was more than 100 μM [16]. Under such a situation, we accidentally found that human hepatoma Li23-derived ORL8c cells, whose gene expression profile was distinct from that of HuH-7 cells, enabling efficient HCV RNA replication and persistent HCV production, had high sensitivity to RBV [16–18]. Therefore, using Li23-derived HCV RNA-replicating cells (ORL8 and ORL11), we demonstrated that RBV at clinically relevant concentrations causes the inhibition of IMPDHs activity, resulting in GTP depletion and the inhibition of HCV replication [16]. Furthermore, we recently demonstrated that adenosine kinase, which phosphorylates RBV to generate mono-phosphorylated RBV, which in turn inhibits IMPDHs, is an essential determinant of anti-HCV activity of RBV in cell culture [19].

Although we have found that adenosine kinase is a crucial factor for ORL8 cells to be sensitive to RBV as mentioned above, we thought that this finding was obtained by the comparison between specific monoclonal cell lines (OR6 and ORL8). Therefore, we hypothesized that there might be other factors determining RBV-sensitivity against HCV RNA replication. To clarify this point, we tried to obtain cells possessing RBV-resistant phenotype from Li23-derived genome-length HCV RNA-replicating OL8 cells [17] possessing an RBV-sensitive phenotype. Here, we report the successful establishment of RBV-resistant OL8-derived cell lines and their characterization.

## Materials and Methods

### Cell cultures

Genome-length HCV RNA-replicating cells (Li23-derived OL8 cells [17]) were maintained in medium for Li23 cells in the presence of 0.3 mg/ml G418 (Geneticin, Invitrogen, Carlsbad, CA) to sustain the efficient replication of HCV RNA as described previously [17]. HCV RNA-replicating cells possess the G418-resistant phenotype, because neomycin phosphotransferase as a selective marker was produced by the efficient replication of HCV RNA. Therefore, when HCV RNA is excluded from the cells or when its level is decreased, the cells die in the presence of G418. To establish RBV-resistant cell lines, we used OL8 cells or OL8 cells that had been passaged every 7 days for 3.5 years (designated as OL8(3.5Y)) [20].

### RBV treatment

For the initial treatment with RBV, cells were plated onto a 10 cm dish (with 60-fold dilutions of confluent cells) and cultured for one day immediately before RBV treatment. RBV (Yamasa, Chiba, Japan) was added to the cells at a final concentration of 100 μM. At 6 days after the initial addition of RBV, cells were passaged with 60-fold dilutions and cultured with RBV (100 μM). Afterwards, when the cells reached a condition of confluence, they were passaged with two-fold dilutions. These cell cultures were continued for 10 weeks with the addition of RBV at 7-day intervals. For further treatment with RBV, cells were plated onto a 10 cm dish (1 x 10^5^ cells/dish) and cultured with RBV by increasing its concentration step by step from 100 μM to 200 μM. To examine the RBV sensitivity of the cells harboring genome-length HCV RNA, the cells were plated onto six-well plates (5 x 10^4^ cells/well) in the medium without G418 and cultured for one day. The cells were then treated with RBV at several concentrations for 3 days. After treatment, the cells were subjected to quantitative reverse transcription-polymerase chain reaction (RT-PCR) analysis for HCV RNA or to Western blot analysis for NS5B as the HCV protein.

### cDNA microarray analysis

Total RNAs from OL8-derived cells were prepared using the RNeasy extraction kit (Qiagen, Hilden, Germany), and we confirmed their ratio of absorbance at 260 over 280 to be from 1.9 to 2.1. As previously described [17, 18, 20], cDNA microarray analysis was performed by Dragon Genomics Center of Takara Bio (Otsu, Japan) through an authorized Affymetrix service provider using the GeneChip Human Genome U133 Plus 2.0 Array. Result of the microarray was deposited in Gene Expression Omnibus (accession number: GSE60948).

### Quantitative RT-PCR

Total RNAs from OL8-derived cells were prepared with an RNeasy extraction kit (Qiagen) or an ISOGEN (Nippon Gene, Tokyo, Japan). The quantitative RT-PCR analysis for HCV RNA was performed using a real-time LightCycler PCR (Roche Diagnostics, Basel, Switzerland) as described previously [15, 17]. Quantitative RT-PCR analysis for the mRNAs of the selected genes was also performed using a real-time LightCycler PCR. The primer sets used in this study are as follows: *RBPMS2*, 5’-tcacctacccaactgccact-3’ and 5’-aagggtaccagcgcacct-3’; *PLA2G7*, 5’-caacggttattcagactcttagtgaag-3’ and 5’-ccagtggaaacatccatgc-3’; *FGA*, 5’-ggaaattttgagaggcgattt-3’ and 5’-cctctgacactcggttgtagg-3’; *IL32*, 5’-caggggagataccatgatcg-3’ and 5’-acggactaatacggcaacaga-3’; *SOX6*, 5’-ttcaggaaagcccatttacc-3’ and 5’-cctcagctgtgctgttcaag-3’; *PRKD1*, 5’-tgtattaccctctttcagaatgaca-3’ and 5’-ccagagacaaaatttcagataaagg-3’; *CYFIP2*, 5’-gccaacgtccagccttatta-3’ and 5’-acgcccttcctgtaggagctgt-3’; *CTBP2*, 5’-gctcaatggtgccacataca-3’ and 5’-tccatggctgcaggaagt-3’; *SOX4*, 5’-agccggaggaggagatgt-3’ and 5’-ttctcgggtcatttcctagc-3’; *TXNIP*, 5’-cttctggaagaccagccaac-3’ and 5’-gaagctcaaagccgaacttg-3’; *CD70*, 5’-ccgtgggaatctgctctc-3’ and 5’-gggaggcaatggtacaacc-3’; *ATP5F1*, 5’-acgtggtgcaaagcatctc-3’ and 5’-tttgccagcagctttaggtc-3’. The relative expression of each gene was normalized by *ATP5F1*.

### Western blot analysis

The cells were harvested with passive lysis buffer (Promega, Madison, WI) according to the manufacturer’s recommendation. After measuring the protein concentration by using the Bio-Rad Protein assay system (Bio-Rad, Hercules, CA), the lysates were mixed with 2x sample buffer (125 mM TrisHCl (pH 6.8), 4% sodium dodecyl sulfate (SDS), 20% glycerol, 10% ß-mercaptoethanol, 0.005% bromophenol blue) and boiled for 5 min. Proteins were separated by SDS-polyacrylamide gel electrophoresis, and immunoblotting analysis was performed as previously described [21]. The antibodies used in this study were mouse anti-NS5B antibody (a generous gift from Dr. Kohara, Tokyo Metropolitan Institute of Medical Science) and mouse anti-ß-actin antibody (Sigma, St. Louis, MO). Immunocomplexes were detected by using a Renaissance enhanced chemiluminescence assay (Perkin Elmer Life Science, Boston, MA).

### Sequence analysis of HCV RNA

Genome-length HCV RNAs were sequenced as previously described [15, 21, 22]. Briefly, to amplify genome-length HCV RNA, RT-PCR using SuperScript II (Invitrogen, Carlsbad, CA) and KOD-plus DNA polymerase (Toyobo, Osaka, Japan) was performed separately in two parts: one part (5.1 kb) covered from the 5’-untranslated region (5’-UTR) to the amino terminal of the NS3 region, and the other part (6.1 kb) covered from the NS2 region to the NS5B region. These fragments overlapped at the NS2 and NS3 regions and were used for sequence analysis of the HCV open reading frame after cloning into pBR322MC [21]. The nucleotide sequences of more than three independent clones obtained were determined.

### Molecular evolutionary analysis

The nucleotide and deduced aa sequences of the clones obtained by RT-PCRs were analyzed by neighbor-joining analysis using the program GENETYX-MAC (Software Development, Tokyo, Japan).

### Preparation of cured cells

To prepare cured cells, HCV RNA-replicating cells were treated with IFN-γ as described previously [17]. Briefly, the cells were treated with IFN-γ (1000 IU/ml) in the absence of G418. The treatment was continued for 3 weeks with the addition of IFN-γ at 4-day intervals. We confirmed that all of the treated cells were dead when cultured in the presence of G418 (0.3 mg/ml) for an additional two weeks. The lack of HCV RNA and protein (NS5B) was also confirmed by quantitative RT-PCR and Western blot analyses of the treated cells, respectively.

### RNA transfection and selection of G418-resistant cells

RNA transfection into the cells was preformed by electroporation as described previously [23]. Briefly, total RNA (100 μg) isolated from the cells was electroporated into 8 x 10^6^ cured cells, and then 1 x 10^5^ or 3 x 10^5^ cells were seeded into a 10 cm diameter dish. After 48h, G418 (0.3 mg/ml) was added and the medium was changed twice per week. After 3 weeks, the G418-resistant colonies obtained on the culture dish were pooled as ployclonal cells and were used for further analysis.

### Statistical analysis

Determination of significance of differences among groups was assessed using the Student’s *t*-test with a two-sided test. *P*<0.05 was considered statistically significant. Data were obtained from three or four independent experiments.

## Results

### Establishment of HCV RNA-replicating cell lines possessing RBV-resistant phenotype

**Fig 1:**
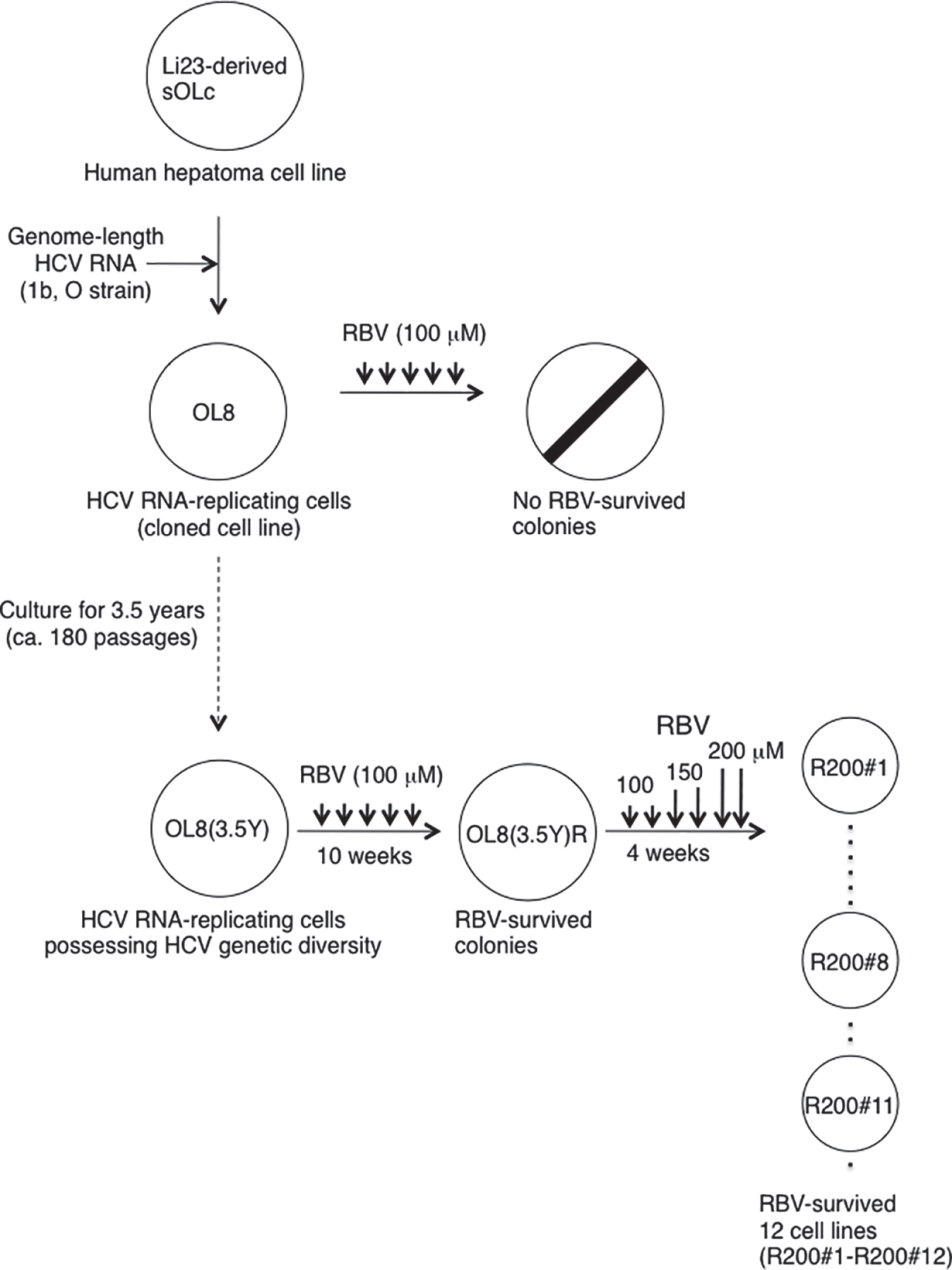
Outline of the isolation of genome-length HCV RNA-replicating cells possessing RBV-resistant phenotype. OL8 or OL8(3.5Y) cells were cultured in the presence of 100 μM RBV for 5 to 10 weeks. The obtained RBV-survived colonies were named OL8(3.5Y)R. The mixed OL8(3.5Y)R cells were further treated with RBV increasingly, step by step, at final concentrations of 100, 150, and 200 μM, resulting in 12 independent RBV-survived colonies (named R200#1 to R200#12). Three colonies, R200#1, R200#8, and R200#11, were further analyzed as representative RBV-resistant cell lines.

To verify the hypothesis that there might still be an unknown mechanism underlying the anti-HCV activity of RBV, we attempted to establish RBV-resistant cell lines from RBV-sensitive HCV RNA-replicating OL8 cells, which were obtained by the transfection of genome-length HCV RNA (O strain of genotype 1b) to Li23-derived sOLc cells [17], using the repetitive treatment with 100 μM of RBV. Unfortunately, however, we failed to obtain the desired cell lines (Fig. 1). Therefore, we decided to next use OL8 cells, which acquired the genetic diversity of the HCV by long-term culture [24]. Thus, using the OL8(3.5Y) cells [20], which had been continuously passaged every 7 days for 3.5 years in the G418-containing medium, we tried to obtain RBV-resistant cells by repetitive RBV treatment. After 10 weeks with RBV treatment repeated every week, we obtained for the first time various sizes of RBV-survived colonies (Fig. 1), suggesting that a small population of parental OL8(3.5Y) cells possessed RBV-resistant phenotype or became RBV-resistant phenotype during 10 weeks of RBV treatment. To obtain cells showing reliable RBV resistance, these survived colonies were pooled once and further treated with RBV using the step-by-step method in 100, 150, and 200 μM for 9, 6, and 15 days, respectively. Consequently, 12 distinct monoclonal cell lines (designated as R200#1 to R200#12), which had survived the treatment with 200 μM of RBV, were obtained (Fig. 1). Among these cell lines, three cell lines (R200#1, R200#8, and R200#11) were selected as representative cells for further characterization, because these cell lines grew up earlier than other cell lines after having moved each cell line to 3.5 cm culture dish. During and after establishment of RBV-resistant cell lines from OL8(3.5Y) cells, G418 was always contained in the medium, except for assaying RBV sensitivity against HCV RNA replication.

Since it was previously reported that cell proliferation affected the efficiency of HCV RNA replication [25], the possibility remains that only the colonies with a growth-rate advantage are able to survive RBV treatment. Thus, we compared the growth rates among OL8(3.5Y), R200#1, R200#8, and R200#11 cells. No growth advantage of R200#1, R200#8, or R200#11 cells was observed compared with OL8(3.5Y) cells (S1 Fig.).

### Evaluation of the effect of RBV on HCV RNA replication in RBV-survived cells

**Fig 2:**
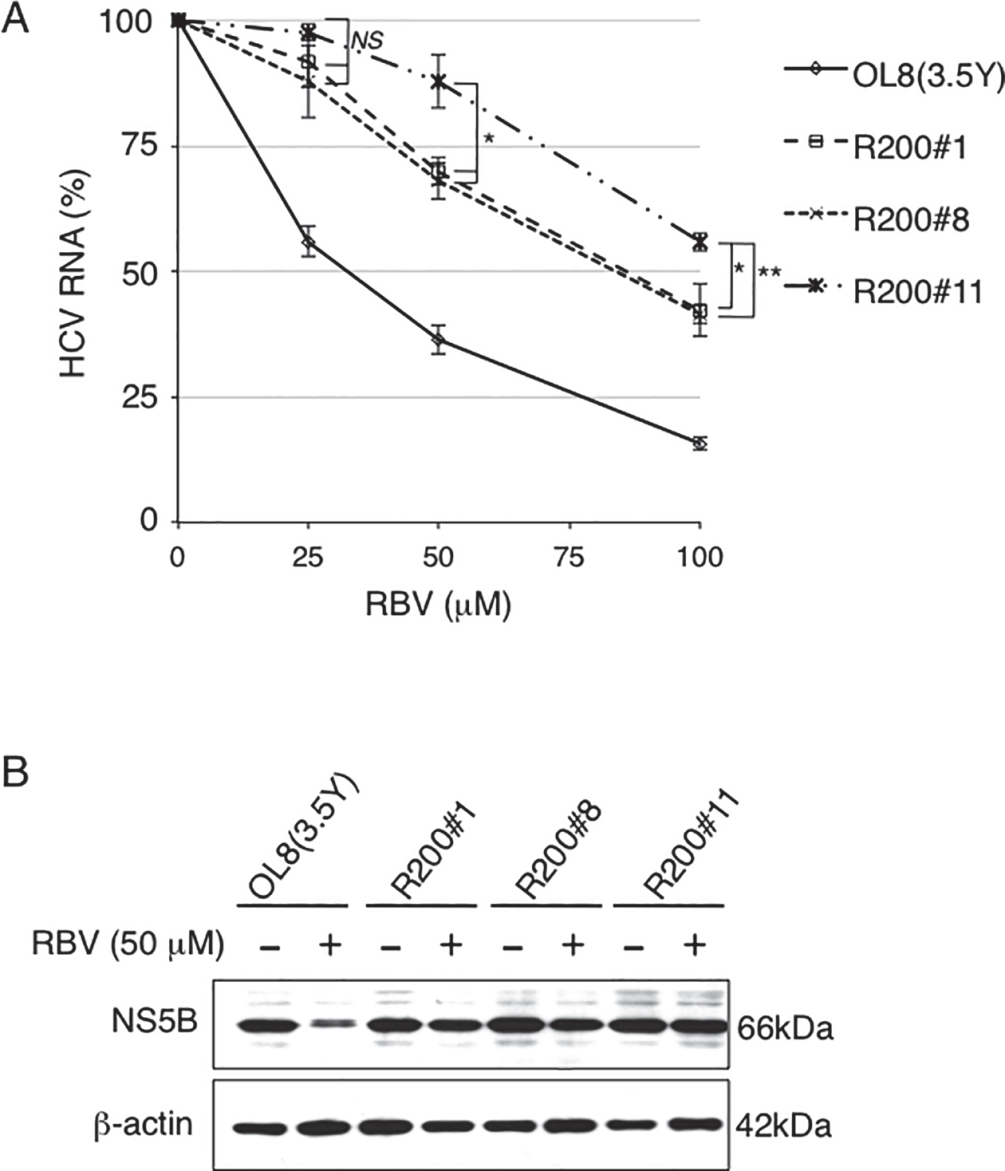
Characterization of OL8(3.5Y)-derived cell lines possessing RBV-resistant phenotype. (A) R200#1, R200#8, R200#11, and OL8(3.5Y) cells were treated with RBV (25, 50, and 100 μM) for 3 days, followed by real-time LightCycler PCR for intracellular HCV RNA. The data are expressed as the means±standard deviation of four independent experiments. Asterisks indicate significant differences compared to R200#11 cells at each RBV concentration. *P<0.05; **P<0.01; *NS*, not significant. The relative value (%) calculated at each point, when the level in nontreated cells was assigned to 100%, is presented here. (B) Production of NS5B in the cells with or without treatment of 50 μM RBV for 3 days was analyzed by immunoblotting using anti-NS5B antibody. ß-actin was used as a control for the amount of protein loaded per lane.

To evaluate the effect of RBV on HCV RNA replication in these newly established cell lines, we examined the levels of HCV RNA and protein in the OL8(3.5Y), R200#1, R200#8, or R200#11 cells treated with RBV for 3 days by quantitative RT-PCR and Western blot analyses, respectively. The level of HCV RNA in the OL8(3.5Y) cells decreased with RBV treatment in a dose-dependent manner, and the EC_50_ value of RBV was 31.1 μM (Fig. 2A and S1 Table), indicating that OL8(3.5Y) cells have an RBV-sensitive phenotype. This result is not inconsistent with a previous result that OL8-derived ORL8 cells showed the RBV-sensitive phenotype [16]. On the other hand, quantitative RT-PCR analysis revealed that R200#1, R200#8, and R200#11 cells showed RBV-resistant phenotype (Fig. 2A and S1 Table). Despite surviving from 200 μM RBV treatment, these R200 cells showed a 40% to 60% reduction in HCV RNA replication by 100 μM RBV treatment, indicating that R200 cells are not completely, but relatively resistant to RBV compared to OL8(3.5Y) cells. The EC_50_ values of RBV in R200#1, R200#8, and R200#11 cells were 83.5, 80.0, and >100 μM, respectively (Fig. 2A and S1 Table). These results revealed that R200#11 cells were more resistant to RBV than R200#1 and R200#8 cells. The significant differences in RBV sensitivity observed between OL8(3.5Y) cells and R200 series cells were also confirmed by Western blot analysis of HCV NS5B (Fig. 2B). Taken together, these results indicate that R200#1, R200#8, and R200#11 cells possess RBV-resistant phenotype.

### Genetic and comparative analyses of HCVs obtained from RBV-resistant cells and parental OL8(3.5Y) cells

**Fig 3:**
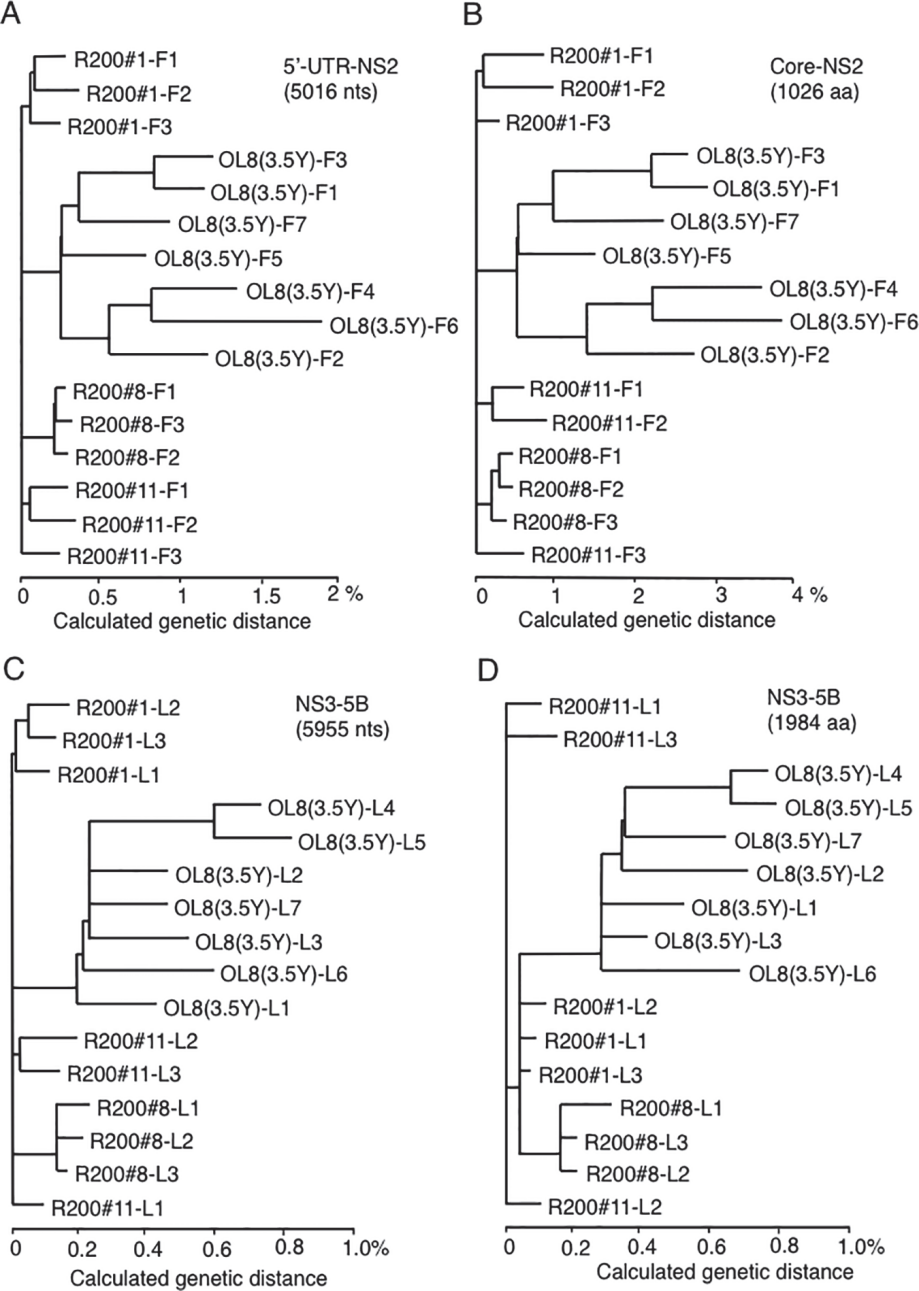
Phylogenetic trees of HCV populations obtained from R200#1, R200#8, R200#11, and OL8(3.5Y) cells. (A) Phylogenetic tree depicted on the basis of nucleotide sequences of the 5’-UTR to NS2 region (5,016 nucleotides). (B) Phylogenetic tree depicted on the basis of deduced aa sequences of Core to NS2 regions (1,026 aa). (C) Phylogenetic tree depicted on the basis of nucleotide sequences of NS3 to NS5B regions (5,955 nucleotides). (D) Phylogenetic tree depicted on the bases of deduced aa sequences of the NS3 to NS5B regions (1,984 aa).

To consider the possibility that genetic variations of HCV led to the acquisition of the RBV-resistant phenotype, we performed sequence analysis of genome-length HCV RNAs obtained from R200#1, R200#8, R200#11, and OL8(3.5Y) cells. The nucleotide sequences of each of three independent cDNA clones in R200 series cells and seven independent cDNA clones in OL8(3.5Y) cells were determined, and these nucleotide sequences were compared with each other. Amino acid sequences deduced from the nucleotide sequences of HCV RNAs were compared between those in the OL8(3.5Y) cells and in each of the R200 series cells. As shown in Table 1, six conserved aa substitutions (mutated in all three clones sequenced in comparison with those in OL8(3.5Y) cells) were found in all R200 series cells (F24L, C172R, S175P, I1641M, V2244A, and T2351A shown by bold letters in Table 1). In addition, several aa substitutions were found in only one or two of R200 series cells (shown by italic letters in Table 1). Phylogenetic tree analysis of HCV species sequenced (5’-UTR to the NS2 region and NS3 to the NS5B regions) revealed that HCVs obtained from R200 series cells were clustered and distinct from the HCVs obtained from OL8(3.5Y) cells at both the nucleotide and aa sequence levels (Fig. 3). These findings indicated that HCV RNAs obtained from the RBV-resistant cells were limited minor populations among the populations of OL8(3.5Y) cells and mutually closely-related populations among three R200 series cells. Taking these results together, we suggest that one or more of these aa substitutions detected could be responsible for the acquisition of RBV-resistant phenotype or for the unique features of each RBV-resistant cell line.

**Table 1:** Amino acid substitutions detected in HCV RNAs obtained from the RBV-resistant cells.

		OL8(3.5Y)	R200#1	R200#8	R200#11
Region	AA position				
Core	10	K(4)R(2)E(1)	R	*G*(2)R(1)	R
	11	T(5)S(2)	S	S	S
	24	F	**L**	**L**	**L**
	104	R	R	*Q*	*Q*(2)R(1)
	172	C	**R**	**R**	**R**
	175	S	**P**	**P**	**P**
E1	264	L	L	*F*	L
E2	422	I(6)V(1)	V	V	V
	460	R	*C*	R	R
p7	787	A	A	*T*	*T*(2)A(1)
NS2	898	P	P	*L*	P
	1097	I(6)T(1)	T	T	T
NS3	1115	P	P	*L*	P
	1445	T	T	*S*	*S*
	1641	I	**M**	**M**	**M**
	1657	T(5)A(2)	A	A	A
NS4B	1962	Q	Q	*H*	Q
NS5A	2244	V	**A**	**A**	**A**
	2310	V	V(2)*M*(1)	*M*	V
	2351	T	**A**	**A**	**A**

The number in parentheses shows the number of indicated amino acids found among seven clones in OL8(3.5Y) or three clones in each R200 series cells. The amino acid without parentheses means that it was found in all clones. Conserved aa substitutions among three R200 cell species are shown by bold letters. Amino acid substitutions found only in one or two R200 cell species are shown by italic letters.

### Comparison of host gene expression profiles between the RBV-resistant cells and the parental OL8(3.5Y) cells

**Fig 4:**
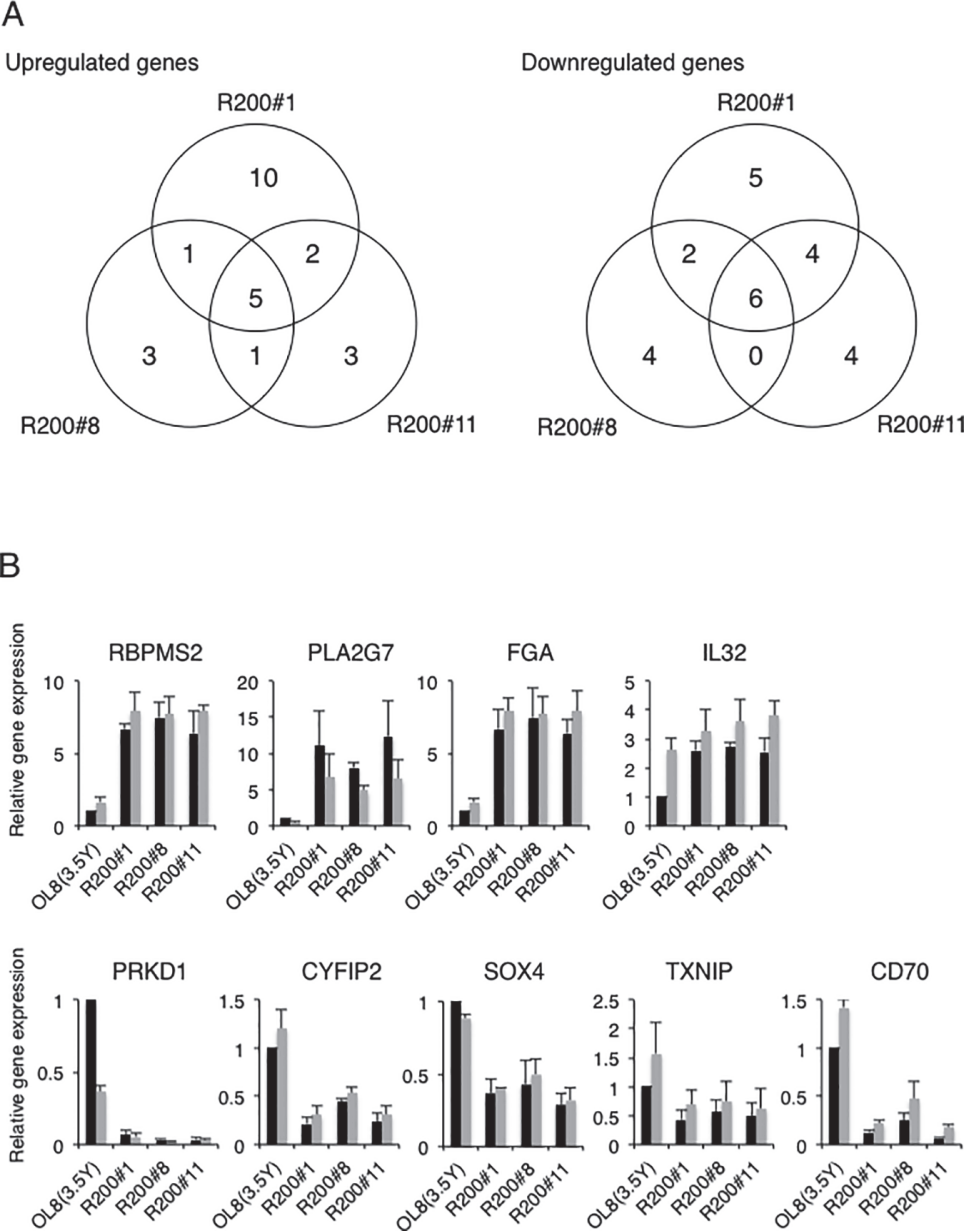
Genes differentially expressed in the RBV-resistant cell lines compared to those in the parental OL8(3.5Y) cell line. (A) Genes whose expression levels were upregulated (left panel) or downregulated (right panel) at ratios of more than 4 or less than 0.25 in the case of each RBV-resistant cells versus OL8(3.5Y) cells were selected. Five genes upregulated and six genes downregulated in all three comparisons were obtained. (B) Expression profiles of representative genes upregulated or downregulated commonly among the RBV-resistant cells. Quantitative RT-PCR analysis was performed using the total RNAs prepared from the cells treated with (gray bar) or without (black bar) 50 μM RBV for 3 days. The data are expressed as the means±standard deviation of three independent experiments. Relative level of each gene expression is shown with assignment to 1 in the nontreated OL8(3.5Y) cells.

To consider the possibility that the alterations of cellular factors are involved in the acquisition of an RBV-resistant phenotype, cDNA microarray analyses were performed using total RNAs prepared from RBV-sensitive OL8(3.5Y) cells and RBV-resistant R200#1, R200#8, and R200#11 cells. To avoid the selection of genes showing low expression, the ratios and expression values were used in combination for selection: genes upregulated more than four-fold with an expression level of more than 500 (actual value of measurement) in each of the RBV-resistant cells or downregulated less than 0.25-fold with an expression level of more than 500 in OL8(3.5Y) cells. By this selection process, we obtained 18, 10, and 11 genes exhibiting upregulation and 17, 12, and 14 genes exhibiting downregulation in R200#1, R200#8, and R200#11 cells, respectively, compared with OL8(3.5Y) cells (Fig. 4A). Adenosine kinase gene, which has been shown to be an RBV-sensitivity determining factor [19], was not selected in these criteria, suggesting that adenosine kinase is not an only factor that affects RBV-sensitivity against HCV RNA replication. Among these genes, five genes (RNA binding protein with multiple splicing 2 [*RBPMS2*], phospholipase A2VII [*PLA2G7*], fibrinogen alpha chain [*FGA*], SRY-box 6 [*SOX6*], and interleukin 32 [*IL32*]) were commonly upregulated, and six genes (protein kinase D1 [*PRKD1*], cytoplasmic FMR1 interacting protein 2 [*CYFIP2*], SRY-box 4 [*SOX4*], C-terminal binding protein 2 [*CTBP2*], thioredoxin interacting protein [*TXNIP*], and CD70 molecule [*CD70*]) were commonly downregulated in RBV-resistant cells. Table 2 summarizes the genes whose expression levels were commonly altered among the three RBV-resistant cell species compared with OL8(3.5Y) cells. To confirm the results of our microarray analysis, quantitative RT-PCR analysis was performed using total RNAs prepared from OL8(3.5Y), R200#1, R200#8, and R200#11 cells (Fig. 4B, black bars). The results revealed that the resultant mRNA expression ratios were mostly correlated with those of microarray analysis, except that of *SOX6* or *CTBP2*, each of which was not detected in our quantitative RT-PCR conditions (Fig. 4B). Among the genes examined in this quantitative RT-PCR, the expression levels of *RBPMS2*, *PLA2G7*, *FGA*, *PRKD1*, and *CD70* were commonly altered by more than four-fold among the three RBV-resistant cell species compared with OL8(3.5Y) cells. We also examined the effects of RBV treatment on the expression of these genes (Fig. 4B, gray bars). We noticed that *IL32* and *PRKD1* in OL8(3.5Y) cells were remarkably upregulated and downregulated, respectively, by RBV treatment. On the basis of these results taken together, we suggest that one or more genes selected by microarray analysis also contribute to the acquisition of RBV-resistant phenotype.

**Table 2:** Genes whose expression levels were commonly altered among R200#1, R200#8, and R200#11 cells compared with OL8(3.5Y) cells.

	Relative mRNA expression ratio		
	R200#1/OL8(3.5Y)	R200#8/OL8(3.5Y)	R200#11/OL8(3.5Y)
Upregulated genes			
RBPMS2	13.1	9.78	9.58
PLA2G7	6.46	6.48	6.98
FGA	5.50	5.47	5.98
SOX6	5.17	4.07	4.53
IL32	4.48	4.35	5.10
Downregulated genes			
PRKD1	0.05	0.06	0.02
CYFIP2	0.09	0.25	0.16
SOX4	0.13	0.18	0.10
CTBP2	0.18	0.03	0.16
TXNIP	0.22	0.22	0.22
CD70	0.25	0.20	0.13

### Exchange analysis of the OL8(3.5Y) and R200#11 cells-derived total RNAs into OL8(3.5Y)c and R200#11c cells

**Fig 5:**
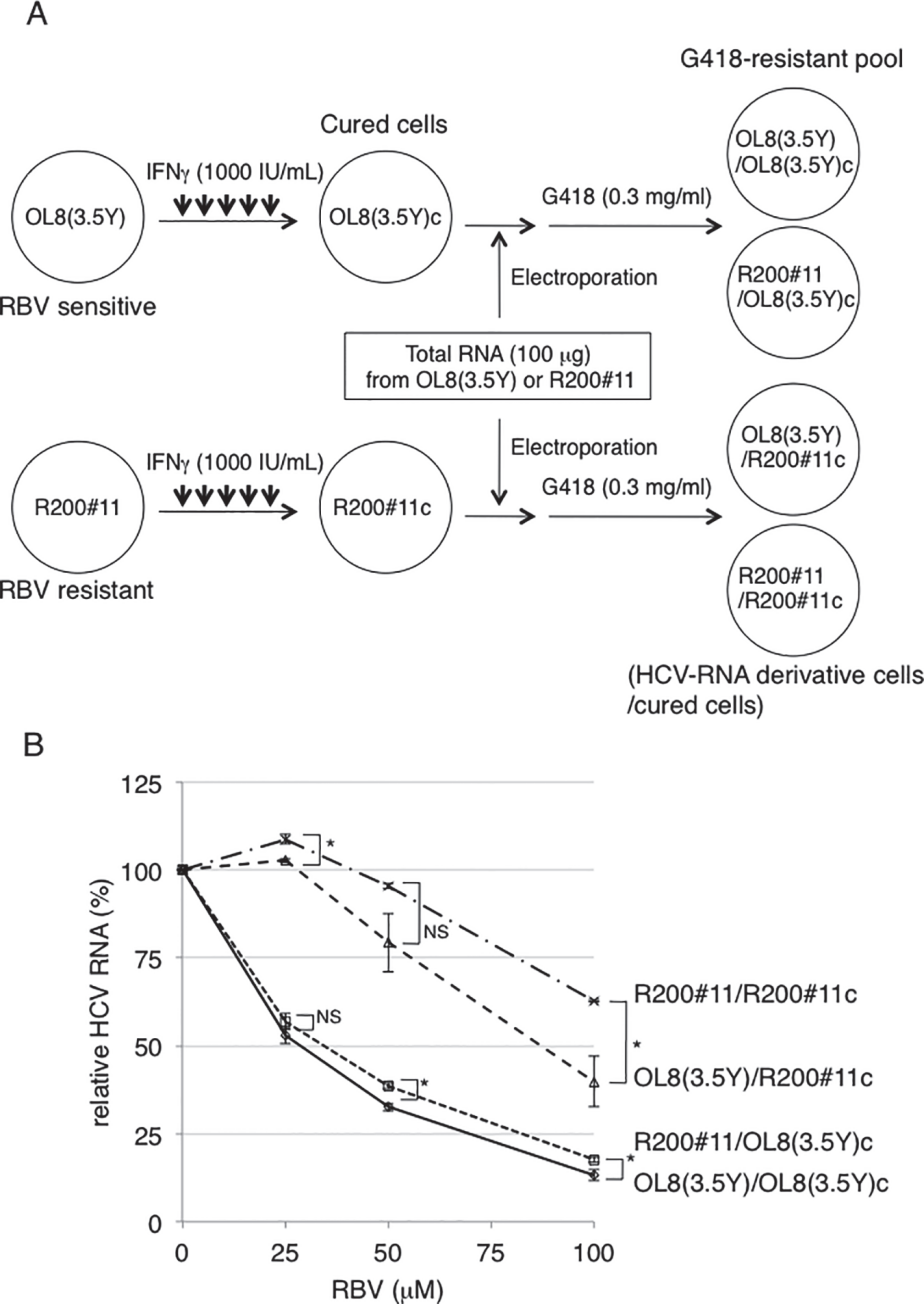
Exchange analysis of the OL8(3.5Y) and R200#11 cells-derived total RNAs into OL8(3.5Y)c and R200#11c cells. (A) Outline of the development of renewed genome-length HCV RNA-replicating cells. Total RNAs (100 μg) derived from OL8(3.5Y) or R200#11 cells, in which approximately 2 x 10^7^ copies of genome-length HCV RNA per 1 μg total RNAs were contained, were electroporated into OL8(3.5Y)c and R200#11c cells. The renewed HCV RNA-replicating cells were selected as polyclonal cells after 2–3 weeks G418 selection. Four cell species obtained were systematically named as HCV RNA-derived cells/HCV RNA-cured cells such as R200#11/OL8(3.5Y)c or OL8(3.5Y)/R200#11c. (B) OL8(3.5Y)/OL8(3.5Y)c, R200#11/OL8(3.5Y)c, OL8(3.5Y)/R200#11c, and R200#11/R200#11c cells, which were maintained in the G418-containing medium for more than 5 weeks after RNA electroporation, were treated with RBV for 3 days and assayed as described in Fig. 2A. The data are expressed as the means±standard deviation of three independent experiments. *P<0.05; *NS*, not significant.

To determine whether viral or host factor contributes to the acquisition of RBV-resistant phenotype based on the effect of RBV on HCV RNA replication, we developed newly four HCV RNA-replicating cell species (designated as OL8(3.5Y)/OL8(3.5Y)c, R200#11/OL8(3.5Y)c, OL8(3.5Y)/R200#11c, and R200#11/R200#11c) by transfection of total RNAs isolated from OL8(3.5Y) or R200#11 cells into cured OL8(3.5Y) (OL8(3.5Y)c) or cured R200#11 (R200#11c) cells (Fig. 5A). Using quantitative RT-PCR analysis as assessed in Fig. 2A, we evaluated the effect of RBV on HCV RNA replication in these developed cells, which had been maintained in G418-containing medium for more than 5 weeks after RNA transfection. The results revealed that the EC_50_ values of RBV in OL8(3.5Y)/OL8(3.5Y)c, R200#11/OL8(3.5Y)c, OL8(3.5Y)/R200#11c, and R200#11/R200#11c cells were 27.4, 32.3, 84.1, and >100 μM, respectively (Fig. 5B and S2 Table). As expected, R200#11/R200#11c cells and OL8(3.5Y)/OL8(3.5Y)c cells showed an RBV-resistant phenotype and an RBV-sensitive phenotype, respectively (Fig. 5B and S2 Table), indicating that RBV-resistant or RBV-sensitive phenotype is reproducibly observed. Regardless of whether HCV RNA was derived from RBV-sensitive OL8(3.5Y) or RBV–resistant R200#11 cells, its replication was more resistant to RBV in R200#11c cells than in OL8(3.5Y)c cells (Fig. 5B and S2 Table), indicating that host factor(s) in R200#11c cells mainly conferred RBV-resistant phenotype. We noticed that replication of HCV RNA derived from R200#11 cells slightly shifted to RBV-resistant phenotype in comparison with that derived from OL8(3.5Y) cells in both host cells, OL8(3.5Y)c and R200#11c (Fig. 5B and S2 Table). These results suggest that RBV-resistant phenotype was conferred mainly by host factor(s) and partially by genetic mutations of HCV RNA.

## Discussion

In this study, we successfully generated RBV-resistant cell lines, in which genome-length HCV RNA replicates, from RBV-sensitive OL8(3.5Y) cells, and characterized representative three lines of them (R200#1, R200#8, and R200#11). By the exchange experiments of the total RNAs derived from OL8(3.5Y) and R200#11 cells, we demonstrated that host factors mainly contributed to the acquisition of RBV-resistant phenotype based on the effect of RBV on HCV RNA replication and viral mutations could be partially involved in determining the degree of RBV resistance. These findings suggest that multiple factors or distinct mechanisms were responsible for the acquisition of RBV resistance.

We first examined the viral factors by the comparative genetic analysis of HCV RNAs obtained from the R200 series cells and the parental OL8(3.5Y) cells. By this analysis, we found six common aa substitutions: F24L, C172R, and S175P in Core; I1641M in NS3; and V2244A and T2351A in NS5A. Among these aa substitutions, I1641M in NS3 and T2351A in NS5A were also detected as the conserved aa substitutions after a 4-year culture of genome-length HCV RNA-replicating OL and OL11 cells, respectively [24], suggesting these aa substitutions occurred regardless of RBV pressure. Therefore, four remaining aa substitutions, F24L, C172R, and S175P in Core and V2244A in NS5A, are considered to have occurred because of the prolonged RBV treatment (approximately 14 weeks). It is also possible that minor HCV populations possessing characteristic aa substitutions survived the prolonged RBV treatment. Indeed, we observed that several additional aa, such as 1097T and 1657A, that were minor populations in OL8(3.5Y) cells, became dominant populations in RBV-resistant cells after RBV treatment. Phylogenetic tree analysis also supported this possibility (Fig. 3). However, we are not able to exclude the possibility that one or more of these aa partly contribute to the acquisition of RBV-resistance or are involved in the distinct resistant phenotype on HCV RNA replication. To clarify this, further comprehensive analysis using genetic mutants of HCV is needed.

On the other hand, cDNA microarray analysis using three RBV-resistant cell lines enabled the selection of dozens of host factors that might participate in RBV-resistant acquisition. Among them, we further selected five and six genes that were commonly upregulated and downregulated, respectively, compared with parental OL8(3.5Y) cells. These selected genes attract attention as the first candidates causing RBV resistance, although multiple molecular mechanisms underlying RBV resistance may be present.

Among these candidates, *PRKD1* and *TXNIP* genes were previously reported to be associated with the regulation of the HCV life cycle. PKD1, a *PRKD1*-encoding protein, is a serine/threonine kinase and has multiple roles in cellular processes, such as cell proliferation, migration, vesicular transport, and differentiation [26]. Recently, Amako et al. demonstrated that HCV secretion, but not HCV RNA replication, is negatively regulated by PKD1 through the phosphorylation of lipid and sterol transfer proteins, CERT and OSBP, which results in the attenuation of the HCV secretion process in the trans-Golgi network [27]. Blackham et al. reported that the antioxidant protein TXNIP was increased along with HCV-JFH-1 infection and was required for both HCV RNA replication and HCV secretion [28]. However, there is thus far no report linking PKD1 or TXNIP to the pathway of the antiviral activity of RBV, such as the inhibition of IMPDH activity or IFN-stimulated-genes induction. To clarify whether altered expression of these genes contributes to the acquisition of RBV-resistant phenotype, it will be necessary to examine the efficiency of HCV RNA replication in the presence or absence of RBV when these genes are knocked down or overexpressed in OL8(3.5Y) or R200 series cells.

Two previous reports showed that ENT1 and CNT3 were responsible for RBV uptake in HuH-7 cells [29], and that ENT1, but not ENT2 or CNTs, was a major RBV uptake transporter in human hepatocytes [30]. As one possible explanation for RBV-resistance of R200 cells may be due to the defectiveness of RBV import in these cells, we compared the expression levels of RBV transporter, ENT1, between OL8(3.5Y) and R200 series cells by quantitative RT-PCR and Western blot analyses. The results revealed no much differences of ENT1 expression among OL8(3.5Y) and three R200 cells in both mRNA and protein levels (S2 Fig.), indicating that the level of ENT1 expression did not associate with the different sensitivity to RBV. However, we can not rule out the possibility that the ENT1 activity for RBV import in R200 series cells is weaker than that in OL8(3.5Y) cells. Therefore, a comparative analysis of ENT1 function among these cells will be also needed.

Regarding the degree of RBV resistance, we noticed that R200#11 cells showed more severe resistant phenotype than R200#1 and R200#8 cells (Fig. 2A and S1 Table). Thus, we examined whether specific viral mutations for R200#11 cells were present; however, viral mutations detected in R200#11 cells were also detected in R200#1 cells, R200#8 cells, or both of them, suggesting that these viral mutations do not contribute to the severe RBV-resistant phenotype. As shown in Fig. 4A, 3 genes and 4 genes were upregulated and downregulated, respectively, more than 4-fold in R200#11 cells specifically in comparison with those in OL8(3.5Y) cells, suggesting that these host factors additively confer RBV-resistant phenotype. We need to examine whether altering the expression levels of these genes affects RBV-sensitivity against HCV RNA replication.

Currently, DAAs for NS3/4A protease, such as telaprevir and boceprevir, are being used to treat chronic hepatitis C [7, 8, 31], and other DAA candidates targeting NS5A or NS5B are waiting for approval [32–34]. However, DAA treatment may cause the emergence of their resistant viruses due to the error-prone polymerase activity of NS5B, eventually leading to the relapse of hepatitis. The combination of DAAs with IFN, RBV, or both is optimal for the therapy, and recent therapy trials have revealed that the addition of RBV to DAAs increased SVR and was associated with low relapse rates [35, 36]. However, there has been an increase in side effects by RBV in combination with DAA, including severe side effects such as skin rash by telaprevir, ageusia by boceprevir, and advanced anemia by telaprevir/boceprevir [7, 8]. Thus, the clarification of RBV’s anti-HCV mechanism and the acquisition mechanism underlying RBV resistance is important to improve the efficacy of RBV treatment and to reduce the side effects of RBV. Our newly established HCV RNA-replicating cell lines possessing the RBV-resistant phenotype will provide new insights toward our understanding of RBV actions and toward the development of new regimens for anti-HCV therapy.

## Supporting Information

S1 Fig Cell growth rates of OL8(3.5Y) and the three R200 series cells. Cells were plated onto 6-well plates (2.5 x 10^4^ cells per well) in triplicate. At 24, 48, 72, and 96 h after plating, cells were detached and collected. Cell growth was assayed by counting cells using a hematocytometer. The data are expressed as the means±standard deviation.
(TIF)

S2 Fig Expression of RBV transporter, ENT1 in OL8(3.5Y) and R200 series cells. (A) Total RNAs were isolated from OL8(3.5Y), R200#1, R200#8, and R200#11 cells and the relative levels of ENT1 mRNA were assessed with quantitative RT-PCR. Primer set for *ENT1* gene was described previously [16]. The data are expressed as the means+standard deviation of triplicate assays. Relative level of ENT1 mRNA normalized by ATP5F1 mRNA is shown with assignment to 1 in OL8(3.5Y) cells. (B) Production of ENT1 protein in the cells was analyzed by immunoblotting using anti-ENT1 antibody [16]. ß-actin was used as a control for the amount of protein loaded per lane.
(TIF)

S1 Table Effect of RBV on HCV RNA replication in OL8(3.5Y) and R200 series cells. (DOC)

S2 Table Effect of RBV on HCV RNA replication in HCV RNA-exchanged cells. (DOC)
