# Supplementary figures and images for: Establishment of Hepatitis C Virus RNA-Replicating Cell Lines Possessing Ribavirin-Resistant Phenotype
Source: PLoS One. 2015 Feb 20;10(2):e0118313. doi: 10.1371/journal.pone.0118313 (PMC4336140; doi:10.1371/journal.pone.0118313)

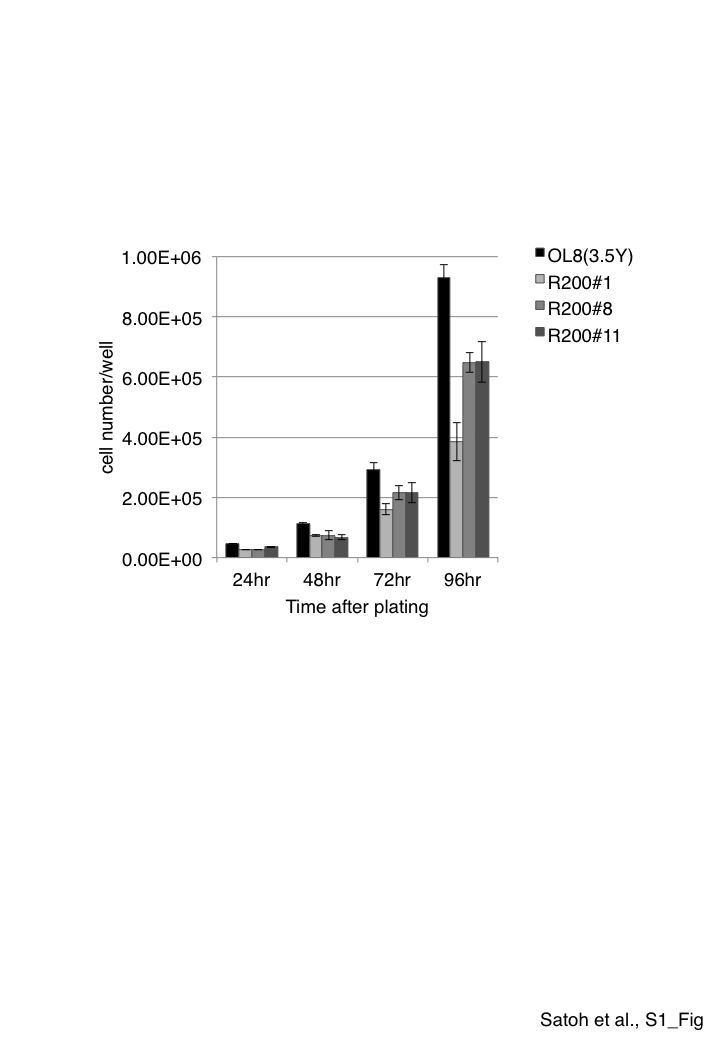

Supplement: S1 Fig — Cells were plated onto 6-well plates (2.5 x 10^4^ cells per well) in triplicate. At 24, 48, 72, and 96 h after plating, cells were detached and collected. Cell growth was assayed by counting cells using a hematocytometer. The data are expressed as the means±standard deviation. (TIF) [file pone.0118313.s001.tif]

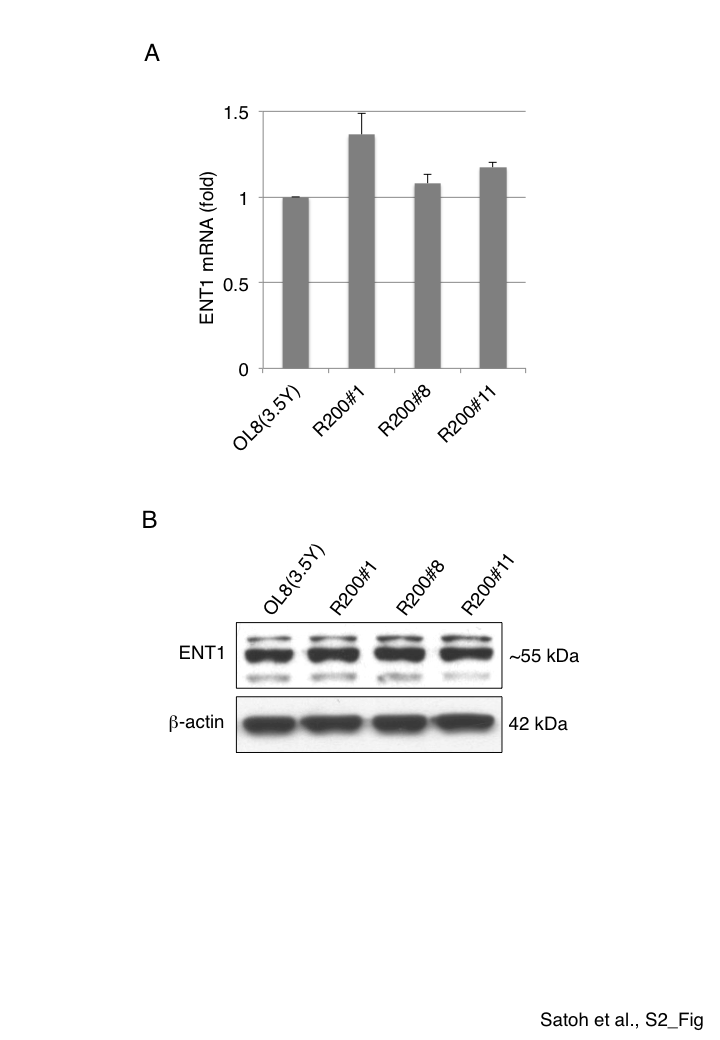

Supplement: S2 Fig — (A) Total RNAs were isolated from OL8(3.5Y), R200#1, R200#8, and R200#11 cells and the relative levels of ENT1 mRNA were assessed with quantitative RT-PCR. Primer set for ENT1 gene was described previously [16]. The data are expressed as the means+standard deviation of triplicate assays. Relative level of ENT1 mRNA normalized by ATP5F1 mRNA is shown with assignment to 1 in OL8(3.5Y) cells. (B) Production of ENT1 protein in the cells was analyzed by immunoblotting using anti-ENT1 antibody [16]. ß-actin was used as a control for the amount of protein loaded per lane. (TIF) [file pone.0118313.s002.tif]
