# Supplementary material for: Establishment of Hepatitis C Virus RNA-Replicating Cell Lines Possessing Ribavirin-Resistant Phenotype
Source: PLoS One. 2015 Feb 20;10(2):e0118313. doi: 10.1371/journal.pone.0118313 (PMC4336140; doi:10.1371/journal.pone.0118313)
Supplement: S1 Table — (DOC) [file pone.0118313.s003.doc]

S1 Table. Effect of RBV on HCV RNA replication in OL8(3.5Y) and R200 series cells.

|  | RBV (μM) | | |
| --- | --- | --- | --- |
| Cells | 25 | 50 | 100 |
| OL8(3.5Y) | 56.0 ± 2.9 | 36.5 ± 2.9 | 15.8 ± 1.3 |
| R200#1 | 92.0 ± 5.4 | 70.0 ± 2.7 | 42.3 ± 5.2 |
| R200#8 | 88.0 ± 7.3 | 68.0 ± 3.7 | 41.5 ± 1.9 |
| R200#11 | 97.8 ± 1.7 | 88.0 ± 5.4 | 56.0 ± 1.8 |

This table shows the results that digitized data of Fig. 2A.

The data are expressed as the means ± standard deviation.

The relative value (%) of HCV RNA calculated at each point, when the level in nontreated cells was assigned to 100%, is presented.
