# Supplementary material for: Establishment of Hepatitis C Virus RNA-Replicating Cell Lines Possessing Ribavirin-Resistant Phenotype
Source: PLoS One. 2015 Feb 20;10(2):e0118313. doi: 10.1371/journal.pone.0118313 (PMC4336140; doi:10.1371/journal.pone.0118313)
Supplement: S2 Table — (DOC) [file pone.0118313.s004.doc]

S2 Table. Effect of RBV on HCV RNA replication in HCV RNA-exchanged cells.

|  | RBV (μM) | | |
| --- | --- | --- | --- |
| Cells | 25 | 50 | 100 |
| OL(3.5Y)/OL8(3.5Y)c | 52.7 ± 2.2 | 32.5 ± 1.1 | 13.1 ± 1.6 |
| R200#11/OL8(3.5Y)c | 56.9 ± 2.3 | 38.5 ± 0.9 | 17.7 ± 0.8 |
| OL8(3.5Y)/R200#11c | 102.6 ± 0.4 | 79.3 ± 8.3 | 39.8 ± 7.1 |
| R200#11/R200#11c | 108.6 ± 1.3 | 95.5 ± 0.8 | 62.7 ± 0.3 |

This table shows the results that digitized data of Fig. 5B.

The data are expressed as the means ± standard deviation.

The relative value (%) of HCV RNA calculated at each point, when the level in nontreated cells was assigned to 100%, is presented.
